# Supplementary material for: EEG state-trajectory instability and speed reveal global rules of intrinsic spatiotemporal neural dynamics
Source: PLoS One. 2020 Aug 27;15(8):e0235744. doi: 10.1371/journal.pone.0235744 (PMC7451514; doi:10.1371/journal.pone.0235744)
Supplement: Attachment — Submitted filename: Response to Reviewers.docx [file pone.0235744.s001.docx]

Reviewer #1: Manuscript summary:

The authors examined scalp EEG during waking quiescence in healthy adult humans. They

characterized large-scale patterns EEG current source density using a pair of novel metrics,

trajectory instability and speed, which describe the evolution of high-dimensional EEG states

over time. Findings include that these quantities vary topographically across the head and when

computed at differing time-scales and that this likely reflects topographical variation in CSD

amplitude spectra. Comparison to phase-scrambled data showed that within- and acrossfrequency

phase alignment affected trajectory instability variably substantially, but had modest

effect when integrated over time. Synchronization results in time averaged reductions in

trajectory speed, while increasing variability similarly to instability. In addition, these metrics are

relatively stable for the (admittedly brief) 5-minute duration of the experiment. Notably, trajectory

instability was found to converge at 100º at a timescale of ~25ms across the entire scalp, a

phenomena warranting further investigation. The authors' thorough controls, including the use of

simulated data and the accounting of volume conduction effects is commendable. As is their

contribution to the sometimes underappreciated observational sciences.

We appreciate these comments.

Minor concerns:

1. In Figure 1C “Unstable” is misspelled “Untable”.

We thank the reviewer for finding this mistake. It has been fixed.

2. If the units of Vk are uV/mm^2, shouldn’t the units of trajectory speed be uV/mm^2·s rather

than a.u.? In general, more explicit specification of units would be welcome.

We agree. As recommended by the reviewer, we now indicate the unit for trajectory speed in

the figures.

3. Subject demographics are listed as male, female, and non-binary. However, male and female

refer to sex, while non-binary refers to gender-identity. Recommended practice[1] would be to

list male, female, and intersex, or men, women, and non-binary (or both); as appropriate.

We agree. We now use the terms, men, women, and non-binary.

4. The precise instructions given to participants before resting-state experiments is a matter of

some debate and may influence brain activity[2]. For these reasons, and to facilitate

reproducibility, could you please include the instructions given in you recording procedures?

We agree. We now provide this information: “Participants were instructed to rest with their eyes

closed while minimizing movement and freely engage in whatever thoughts that spontaneously

arise.” We also mention the article that the reviewer helpfully brought to our attention

demonstrating different fMRI BOLD patterns depending on mind-wandering versus no-thoughts

instructions. It is likely that people would naturally engage in mind wandering when instructed to

rest with their eyes closed unless they are specifically instructed to engage in no thoughts

(presumably attempting thought suppression). To indirectly address the issue of reproducibility,

we have doubled the sample size from *N*=24 to 48.

5. Footnotes are not permitted in PLOS publications. Please incorporate them into the text.

We have incorporated all footnotes into the main text.

References:

1. Clayton JA, Tannenbaum C. Reporting sex, gender, or both in clinical research?. Jama. 2016

Nov 8;316(18):1863-4.

2. Kawagoe T, Onoda K, Yamaguchi S. Different pre-scanning instructions induce distinct

psychological and resting brain states during functional magnetic resonance imaging. European

Journal of Neuroscience. 2018 Jan;47(1):77-82.

Reviewer #2: The authors examined EEG resting state activity in healthy subjects aiming at the

identification of quantitative principles governing large-scale neural dynamics. To do so, they

divided the overall number of electrodes into three sub-networks corresponding to areas

responsible for different cognitive functions. The paper is a methodological study - within the

network science framework – aiming at the identification of relevant global signatures of the

EEG signal. Unfortunately, it is not reported whether the practice of subdividing the EEG resting

state signal within three subnetworks is novel, and at the same time the authors do not clarify

what they mean by global signature of neural dynamics.

We understand the reviewer’s concern that we did not provide sufficient justification for the

method of dividing the electrode space (i.e., the current-source space) into the posterior,

central, and anterior regions for our analyses. We also understand that it was confusing to the

reviewer what we meant by “global signature” especially given that we divided the currentsource

space into three regions. It was not the case that we considered these three regions to

be novel or special in any particular way. We performed comparative analyses across these

regions because many prior studies have reported localization of EEG activity within these

regions depending on the sensory modality of stimuli, type of motor response, attention and

cognitive manipulations, and so on. We cite representative studies. Further, our recent study

(Menceloglu, Grabowecky, & Suzuki, 2020) showed that EEG cross-frequency interactions have

distinct patterns in these three regions as well as in the left-lateral and right-lateral regions. We

have included the left/right-lateral regions in the analyses presented in the revision.

The reason why we examined EEG state trajectories in separate regions that likely mediate

distinct computations was that we wanted to identify dynamical parameters that were globally

preserved. Our strategy of identifying global rules of neural dynamics was to compare dynamics

in regions that likely perform distinct computations to identify dynamical properties that are

globally preserved across those regions. We hope the reviewer finds that this point is clearly

explained in the revision.

The general feeling is that a comparison with the state of the art of the literature in some of the

domains is lacking. Arguments that should justify the choices of the authors are missing. For

comparison, some examples concerning cross-frequency coupling and mind-wondering

(https://www.sciencedirect.com/science/article/abs/pii/S1364661310002068

works from Smallwood et al.)

The authors did summarize the main research question, however it is not clear how their key

findings apply, i.e. which are the main applications of the authors’ research.

The authors should specify what they mean by quantitative principles and, whether they refer to

the quantities in page 4, they should provide clear examples about how informative are those

quantities.

Furthermore, extracting features from the whole scalp EEG signal is a practice in the entire BCI

literature. The whole literature is ignored, while it provides a real-time analysis of EEG features

comparable with those the authors aim at defining in this article. If the authors aim at extracting

intrinsic dynamics during spontaneous thoughts, it is not clear why do they subdivide the

electrodes within 3 regions instead of looking at the whole brain connectivity. This is something

that should be clarified or shifted in the methods section.

We agree that we did not clearly explain how the goals of the current study are distinct from

those of many other studies that employed spectral-decomposition approaches to examine

spatial patterns of within-frequency and cross-frequency interactions measured as amplitudeamplitude,

phase-phase, and phase-amplitude coupling. In fact, we have also recently used a

spectral-decomposition approach to examine cross-frequency interactions (Menceloglu et al.,

2020).

One of the primary reasons for conducting the current study was to examine large-scale neural

dynamics without performing spectral decomposition to provide new/alternative insights, but in

such a way that those insights would be relatable to known oscillatory activity, synchronization,

and waveform characteristics. This is the reason why we chose to examine the timescale

dependence of EEG-state trajectory instability (approximately curvature) and speed (rate of

change). As we describe in the Results and Discussion and General Discussion sections, our

results provide new insights such as a globally convergent level of trajectory instability on the

timescale of ~25 ms, relatively timescale invariant contributions of cross-site within-frequency

and cross-frequency interactions to the temporal average and variability of trajectory instability

and speed, and the general control of global dynamics to reduce synchronized oscillatory

activity. To our knowledge, none of the prior studies using spectral-decomposition approaches

have provided these insights. Further, we have more confidence in our results now that we have

doubled the sample size, examined two additional regions, and included new analyses

suggesting that the coordination of cross-site within-frequency phase synchronization and the

coordination of cross-frequency phase relations independently contribute to similar effects.

We agree with the reviewer that “mind wandering” typically occurs while people rest with their

eyes closed. In fact, some degree of mind wandering occurs no matter what people do. The

potential relevance of mind wandering was also noted by the other reviewer who brought to our

attention an fMRI study demonstrating the engagement of different brain networks depending on

the instructions given to participants while they rested (mind wander vs. no thoughts

instructions). Our instructions were general, essentially telling our participants to rest with their

eyes closed while engaging in any thoughts that may spontaneously arise; thus, it is unclear the

degree to which what our participants did differed from mind wandering. This caveat is now

provided in the Materials and Methods section. Nevertheless, the strong cross-participant

consistency of our results suggests that the dynamical rules that we have identified are

relatively independent of the degree to which people engage in mind wandering or other

cognitive activity while resting with their eyes closed. We are planning future studies to

investigate how the dynamical rules identified in the current study adjust to specific perceptual,

attentional, and cognitive operations.

We believe that the goal of the current study is distinct from the goals of BCI research. The

primary goal of BCI research (as we understand it) is to identify the features of EEG, MEG, or

fMRI-BOLD activity that are most effective for translating mental activity or mental states to

desired output to control computer interfaces. For that purpose, PCA and/or ICA are often

applied to EEG/MEG signals (raw or spectrally decomposed signals) to extract linearly

separable and/or non-linearly orthogonal features that would likely carry the critical information

for controlling the interface in a desired manner. Multivariate analyses (e.g., SVM, CNN) may be

subsequently used to fine-tune those features. In essence, BCI research seeks to identify

EEG/MEG features that are effective for translating specific neural activity to desired control of a

computer algorithm. In this sense, BCI research is closely related to research that seeks to

identify neural correlates of motor behavior, attention, and cognition. The goal of our study was

complementary. We did not seek to identify EEG signals that were most closely associated with

specific behavior or cognition. We sought to identify dynamical rules that globally characterized

large-scale neural activity (accessible to EEG) while people rested with their eyes closed. We

have attempted to more clearly articulate the scientific goals of the current study in the revision.

Methods section:

The authors should clarify how artifacts have been removed. Authors should justify according to

the state of the art why did they apply the Laplacian transform

(https://www.ncbi.nlm.nih.gov/pubmed/18347966), instead of other forms of correction for the

references.

Regarding our procedure for EEG pre-processing, based on our inspection of the EEG data, we

did not find any reasons for removing EEG signals over any time segments, for any electrodes

or from any participants. This is stated in the revision. Further, to address the reproducibility

issue raised by the other reviewer, we doubled the sample size from *N*=24 to 48, allowing us to

more rigorously present individual differences.

The article cited by the reviewer discusses average referencing of EEG in the context of

“microstate” analysis. Microstate analyses have been used to identify a small set of highly

spatially variable instances of EEG activity as representative neural microstates, with the goal of

classifying EEG spatiotemporal activity as a time sequence of a relatively small set of those

microstates. We aimed to extract the neural-source information from scalp recorded EEG in the

most assumption-free (data driven) manner by approximating the spatial distribution of the

underlying neural current sources using a surface-Laplacian transform. The validity of this

procedure is discussed in numerous published articles including the ones we cited (e.g., Kayser

and Tenke, 2012, and an advanced EEG analysis textbook by Cohen, 2014). We want to

characterize the rules of global neural dynamics in terms of multidimensional state trajectories

of the estimated current sources while minimizing volume conduction effects. To our knowledge,

neither average referencing nor procedures related to identifying EEG microstates reduces the

influences of volume conduction or help localize the underlying neural sources.

The authors should clarify why the signal was segmented differently depending on the

participant

This must be a misunderstanding because we divided EEG signals from all participants into

sequential 5-second segments. It is the case that the total number of 5-second segments were

58 for some participants and 59 for the remaining participants because of the slight differences

in the total recording duration across participants. To avoid this confusion, because all analyses

were performed on the first 58 segments obtained from all participants, we now state that we

obtained 58 segments from all participants.

• 0.5 Hz is a frequency reasonable for resting state study. The claim that 5s is an amount of time

which would allow comparison with cognitive tasks is not entirely true, as when it comes for

visual task every trial last up to 500ms. The authors should therefore clarify with what type of

cognitive tasks they are comparing their results to.

We agree that we should have more clearly explained the choice of 5 seconds as the time

segments of our analyses. We could have analyzed the entire 5-minute EEG signals without

segmenting them and would have obtained the same results (stated in the revision). This can be

inferred from the fact that the characteristic patterns measured for successive 50-second

intervals were relatively stable over the 5-minute period (discussed in the revision). We agree

that we could examine our data in time segments shorter than 5 seconds. However, we feel that

5 seconds is appropriate because the longest analysis-timescale that we wanted to include was

about a quarter of a second which corresponds to about a half-second cycle for relevant

underlying oscillatory activity. We wanted each time segment to include a substantial number of

oscillatory cycles for the longest analysis-timescale; we have ~10 cycles within each 5-second

segment. Further, although we agree that some trials in typical psychophysical experiments are

short (e.g., 500 ms as noted by the reviewer) especially if they only include single-stimulus

presentations, a trial may last several seconds including the initial fixation period, stimulus

presentation(s) with ISI(s), and response time. Finally, the choice of 5 second was partly

motivated by our recent finding that a half cycle of several seconds is a characteristic rate at

which EEG spectral amplitudes fluctuate (Menceloglu et al., 2020).

We are a little unsure about how the reviewer’s statement, “0.5 Hz is a frequency reasonable for

resting state study,” is relevant to the length of our analysis time segments. Does the reviewer

wonder whether the rules we identified using 5-second time segments would remain unchanged

if we used 2-second (a period of 0.5 Hz oscillations) time segments? The use of 2-second

instead of 5-second time segments would not make qualitative difference in the results because

(1) the reported results are averaged across all time segments and (2) the boundaries of

consecutive time segments would not be time-locked to any specific EEG activity. The use of 2-

second time segments would make the measures on longer timescales less reliable due to the

inclusion of fewer cycles of relevant oscillations within each time segment (e.g., ~4 cycles

instead of ~10 cycles for the longest timescale we examined). Nevertheless, we see that if one

wished to determine how the rules identified here may rapidly adjust within, for example, 500 ms

of visual onset, it would be useful to repeat all the analyses presented here using 500 ms time

segments to determine the range of timescales for which the intrinsic rules could be reliably

measured within 500 ms. Nevertheless, we think that characterizing how all the rules might

gradually dissipate when shorter time segments were used would be outside the scope of this

study. The goal of the current study was to identify simple, robust, and stable global rules of

EEG spatiotemporal dynamics with decent levels of reliability across a broad range of

timescales using sufficiently short time segments so that the phase-scrambled controls closely

matched the general rate of spectral power fluctuation (Menceloglu et al., 2020).

• The authors should also comment on the settings of the filters of the hardware system used

(Biosemi)

We provide this information now.

• The authors should clarify why they divided in three relevant networks: if the practice is new,

then they should state why it’s relevant – comparing with previous literature. If the practice is not

new, then they should cite other studies that did the same.

Please see our response above. To reiterate, we did not intend to place any significance on the

particular choice of three regions except that they likely reflect distinct underlying computations

based on the general literature and our own recent study. Our goal was to identify general

dynamical parameters that are globally preserved across regions that subserve different

processes. Nevertheless, to indirectly respond to the reviewer’s concern and to increase the

generalizability of our results (especially in light of our recent study showing that lateral regions

display distinct patterns of cross-frequency interactions; Menceloglu et al., 2020), we included

the left-lateral and right-lateral regions in addition to the original three regions in our assessment

of inter-region convergence and invariance of the behaviors of trajectory instability and speed.

Results and Discussion:

The discussion is not structured systematically, according to the findings. Results are not

commented and it is very difficult to distinguish between the original contribution of the article

and previous literature on the topic.

We appreciate this concern. We hope the reviewer finds the descriptions of the current and prior

results to be clearly distinguished in the revision.

I recommend a check of the literature according to the mentioned points. The authors should

revise not the written English, but clarify the meaning of technical terms used now and then,

such as quantitative signature. They should also avoid the usage of ambiguous terms.

We appreciate these recommendations. We hope the reviewer finds that the terms and ideas

are more clearly defined, described, and explained in the revision.
